# Evaluation of commercially available fully automated and ELISA-based assays for detecting anti-SARS-CoV-2 neutralizing antibodies

**DOI:** 10.1038/s41598-022-21317-x

**Published:** 2022-11-08

**Authors:** Hadeel T. Zedan, Hadi M. Yassine, Duaa W. Al-Sadeq, Na Liu, Hamda Qotba, Eleonora Nicolai, Massimo Pieri, Sergio Bernardini, Laith J. Abu-Raddad, Gheyath K. Nasrallah

**Affiliations:** 1 Department of Biomedical Science, College of Health Sciences, QU Health, Qatar University, P.O. Box 2713, Doha, Qatar; 2 Biomedical Research Center, Qatar University, P.O. Box 2713, Doha, Qatar; 3 College of Medicine, QU Health, Qatar University, P.O. Box 2713, Doha, Qatar; 4 Shenzhen Mindray Bio-Medical Electronics Co., Ltd., Shenzhen, China; 5 Department of Clinical Research, Primary Health Care Centers, Doha, Qatar; 6 Department of Experimental Medicine, University of Rome Tor Vergata, 00133 Rome, Italy; 7 Clinical Biochemistry, Tor Vergata University Hospital, 00133 Rome, Italy; 8 Infectious Disease Epidemiology Group, Weill Cornell Medicine-Qatar, Cornell University, Qatar Foundation–Education City, Doha, Qatar; 9 World Health Organization Collaborating Centre for Disease Epidemiology Analytics on HIV/AIDS, Sexually Transmitted Infections, and Viral Hepatitis, Weill Cornell Medicine–Qatar, Cornell University, Qatar Foundation–Education City, Doha, Qatar; 10 Department of Healthcare Policy and Research, Weill Cornell Medicine, Cornell University, New York, USA

**Keywords:** Immunological techniques, Immunology

## Abstract

Rapid and accurate measurement of the severe acute respiratory syndrome coronavirus 2 (SARS-CoV2)-specific neutralizing antibodies (nAbs) is paramount for monitoring immunity in infected and vaccinated subjects. The current gold standard relies on pseudovirus neutralization tests which require sophisticated skills and facilities. Alternatively, recent competitive immunoassays measuring anti-SARS-CoV-2 nAbs are proposed as a quick and commercially available surrogate virus neutralization test (sVNT). Here, we report the performance evaluation of three sVNTs, including two ELISA-based assays and an automated bead-based immunoassay for detecting nAbs against SARS-CoV-2. The performance of three sVNTs, including GenScript cPass, Dynamiker, and Mindray NTAb was assessed in samples collected from SARS-CoV-2 infected patients (n = 160), COVID-19 vaccinated individuals (n = 163), and pre-pandemic controls (n = 70). Samples were collected from infected patients and vaccinated individuals 2–24 weeks after symptoms onset or second dose administration. Correlation analysis with pseudovirus neutralization test (pVNT) and immunoassays detecting anti-SARS-CoV-2 binding antibodies was performed. Receiver operating characteristic (ROC) curve analysis was generated to assess the optimal threshold for detecting nAbs by each assay. All three sVNTs showed an excellent performance in terms of specificity (100%) and sensitivity (100%, 97.0%, and 97.1% for GenScript, Dynamiker, and Mindray, respectively) in samples collected from vaccinated subjects. GenScript demonstrated the strongest correlation with pVNT (*r* = 0.743, R^2^ = 0.552), followed by Mindray (*r* = 0.718, R^2^ = 0.515) and Dynamiker (*r* = 0.608, R^2^ = 0.369). Correlation with anti-SARS-CoV-2 binding antibodies was variable, but the strongest correlations were observed between anti-RBD IgG antibodies and Mindray (*r* = 0.952, R^2^ = 0.907). ROC curve analyses demonstrated excellent performance for all three sVNT assays in both groups, with an AUC ranging between 0.99 and 1.0 (*p* < 0.0001). Also, it was shown that the manufacturer's recommended cutoff values could be modified based on the tested cohort without significantly affecting the sVNT performance. The sVNT provides a rapid, low-cost, and scalable alternative to conventional neutralization assays for measuring and expanding nAbs testing across various research and clinical settings. Also, it could aid in evaluating actual protective immunity at the population level and assessing vaccine effectiveness to lay a foundation for boosters' requirements.

## Introduction

Serological tests are essential to address the coronavirus disease 2019 (COVID-19) pandemic^1^. However, the extent to which positive results from a serological test reflect a protective immunity is still unclear despite their vital role. As described in the interim guidelines for COVID-19 antibody testing published by the Center for Disease Control and Prevention (CDC)^2^, the currently available serological tests measure: (1) binding antibodies (bAbs) targeting various SARS-CoV-2 antigens; (2) functional antibodies (or neutralizing antibodies, nAbs), which neutralize the virus (live or pseudovirus) by targeting the spike (S) protein; or (3) surrogate virus neutralization tests (sVNT) that rely on a competitive approach to assess anti-receptor-binding domain (RBD) antibodies^3^. The most common antigenic targets employed by these assays include the nucleocapsid (N) protein, S protein, the S1 subunit and the RBD^4^.

Although many serological assays, such as enzyme-linked immunosorbent assay (ELISA) and lateral flow assay (LFA) rapid tests, are commercially available for detecting anti-SARS-CoV-2 antibodies, they cannot distinguish bAbs from nAbs. Two assays are considered the gold standard for detecting nAbs: microneutralization assay (MNA) and pseudovirus neutralization test (pVNT)^5^. However, both platforms are research laboratory-based, with MNA requiring the use of specialized biosafety containment facilities and the use of live pathogens. On the other hand, pVNT is relatively inexpensive and not available in many labs across the globe. Hence, both assays are unsuitable for mass production or testing on a commercial scale, even in the most developed nations^6,7^. Consequently, rapid, and reliable high-throughput neutralization assays are needed to detect nAbs in different settings and populations. Several studies have assessed the correlation between serological tests detecting bAbs with neutralizing activity to provide insights regarding the functional capabilities of the detected antibodies^4,8–10^. Expectedly, assays targeting SARS-CoV-2 S protein, particularly the RBD, showed the best correlation with neutralizing activity, indicating that these assays could serve as reliable and high-throughput assays for predicting the presence of nAbs^5^.

Despite the excellent performance and correlation with neutralizing activity seen by the assessed commercial serological tests, direct detection of nAbs is still crucial. Hence, the extent to which positive results by serology reflect a protective immune response is still insufficient for assessing protective immunity. Fortunately, since the COVID-19 pandemic, several commercial immunoassays have been developed to serve as surrogate assays for MNA and pVNT^7^. In this study, we evaluated the performance of three different sVNTs, including GenScript sVNT (cPass), Dynamiker sVNT, and Mindray NTAb sVNT, in comparison to the pVNT. All three assays detect nAbs targeting SARS-CoV-2 RBD by mimicking the virus-host interaction in an ELISA plate-based or bead-based test. This interaction can be blocked by specific nAbs in the patient's sera in the same manner as in MNA or pVNT.

## Methods

### Study design, ethical approval, and clinical samples

The performance of the three assays was assessed using pre-pandemic sera samples (n = 70), convalescent sera collected from COVID-19-recovered patients during the period of SARS-CoV-2 wild-type predominance (April–August 2020) (n = 63 for GenScript sVNT, n = 127 for Dynamiker sVNT, n = 160 for Mindray NTAb, n = 72 for pVNT), and samples collected from individuals vaccinated with COVID-19 mRNA vaccines, either BNT162b2 or mRNA-1273, (n = 45 for GenScript sVNT, n = 137 for Dynamiker sVNT, n = 163 for Mindray NTAb, and n = 77 for pVNT). Samples from either SARS-CoV-2 infected or COVID-19 vaccinated individuals were collected 2 to 24 weeks post symptoms onset/positive RT-PCR test or post-second dose administration, respectively. In addition, the pre-pandemic sera samples were collected from healthy blood donors before 2019. Details about the collection, transport, and storage methods of the control samples were described in previous studies^11–15^. Further details about the demographics and clinical characteristics of the samples are shown in Table 1. To exclude seronegative samples, all samples were tested for anti-SARS-CoV-2 bAbs using the CL-900i^®^ automated analyzer IgG against S and N proteins. Ethical approvals were obtained from the Institutional Review Board at Qatar University (QU-IRB 1537-FBA/21 and QU-IRB 1492-E/21) and the Primary Health Care Corporation (PHCC/DCR/2020/05/047). The study was conducted following the ethics review board's guidelines and regulations. Informed consent was obtained from all study participants.

**Table 1:** Demographic and clinical characteristics of samples collected from SARS-CoV-2 infected cohort (n = 127), vaccinated cohort (n = 121), and controls (n = 70).

	SARS-CoV-2 infected patients	COVID-19 vaccinated individuals	Pre-pandemic controls
**Median age in years (IQR)**	35.0 (27.0–43.5)	35.0 (25.5–44.5)	43.0 (37.0–51.0)
**Gender**			
Female	20 (12.5)	68 (41.7)	3 (4.3)
Male	137 (85.6)	82 (50.3)	67 (95.7)
Not determined	3 (1.9)	13 (8.0)	–
**Disease status**			
Symptomatic	56 (35.0)		
Asymptomatic	90 (56.3)		
Not determined	14 (8.8)		
**Vaccine type**			
Pfizer (BNT162b2)		97 (80.2)	
Moderna (mRNA-1273)		24 (19.8)	
**Median days of sampling (IQR)**^**a**^	46 (20–163)	63 (35–114)	
Total	160	163	70

^a^Median days of sampling post symptoms onset or RT-PCR positive test for SARS-CoV-2 infected cohort or post-second dose administration for the vaccinated cohort. SARS-CoV-2: severe acute respiratory syndrome virus 2, COVID-19: Coronavirus Disease 2019.

*IQR* interquartile range.

### Serological testing

The initial serological screening was done on the automated analyzer CL-900i® from Mindray Bio-Medical Electronics^8,9,16^ using three chemiluminescence immunoassays to detect anti-SARS-CoV-2 bAbs targeting either the S and N proteins or exclusively the RBD: (i) Anti-SARS-CoV-2 IgG against S/N protein (Cat. No. SARS-CoV-2 IgG121) with a cutoff index of ≥ 10 IU/ml, (ii) Anti-SARS-CoV-2 S-receptor binding domain (S-RBD) IgG (Cat. No. SARS-CoV-2 S-RBD IgG122, Mindray, China) with a cutoff index of ≥ 10–1000 BAU/ml, and (iii) Anti-S-RBD SARS-CoV-2 total antibodies (IgG, IgA, and IgM) (Cat. No. SARS-CoV-2 Total Antibodies 122, Mindray, China) with a cutoff index of ≥ 10–2000 AU/ml. All plasma samples were tested with these assays following the manufacturer's instructions.

### ELISA-based surrogate virus neutralization tests (sVNT)

**Figure 1:**
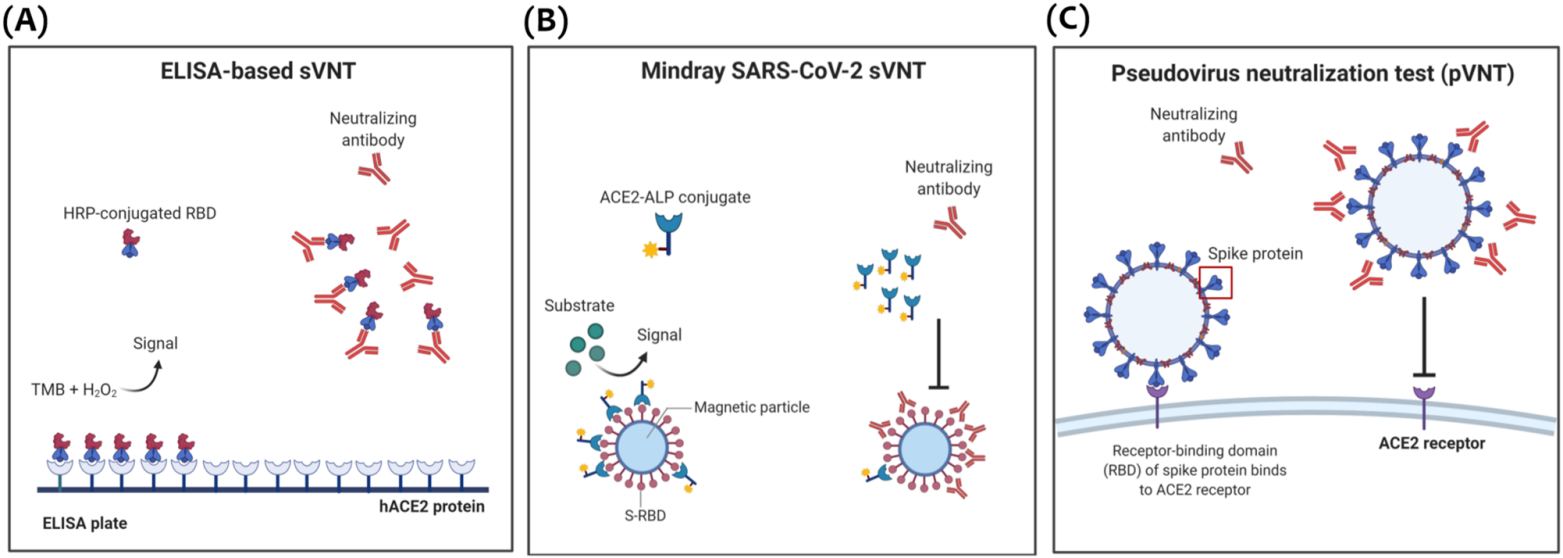
Graphical illustration for the principle of sVNT in comparison to pVNT. (**A**) Principle of ELISA-based surrogate virus neutralization test (sVNT) where anti-SARS-CoV-2 nAbs block the binding of HRP-conjugated RBD protein to the precoated hACE2 protein on the ELISA plate. (**B**) Principle of Mindray competitive binding NTAb immunoassay where anti-SARS-CoV-2 nAbs compete with the ACE2-ALP conjugate for RBD-binding sites on the magnetic beads. (**C**) Mechanism of pseudovirus neutralization test (pVNT) where anti-SARS-CoV-2 nAbs block the binding of SARS-CoV-2 spike (S) protein to human ACE2 receptor on the host cell surface. All illustrations were created using BioRender. Figures (**A**) and (**C**) were adapted from Wang et al.^7^.

Two different SARS-CoV-2 sVNTs were assessed for detecting nAbs that block the interaction between SARS-CoV-2 RBD and human angiotensin-converting enzyme 2 (ACE2) receptors. The first assay is an ELISA-based inhibition test developed by GenScript (Cat. No. L00847, GenScript Biotech, NJ, USA)^7,8,17^. This assay utilizes the same principle as ELISA, using a 96-well microplate to serologically screen for nAbs targeting SARS-CoV-2 RBD (Fig. 1A). The performance of this assay was previously assessed by several studies demonstrating high sensitivity, specificity, and correlation with MNA and pVNT^7,18,19^. Absorbance was measured at 450 nm, and the percentage of inhibition was calculated using the following formula: % inhibition = (1 − (OD450 sample/OD450 of negative control)) × 100. Result interpretation was as follows: percent inhibition ≥ 30% is positive (detectable nAbs), and < 30% percent inhibition is negative (non-detectable nAbs). The second ELISA-based sVNT, developed by Dynamiker Biotechnology (Cat No. DNK-2102-2, Tianjin, China)^20^, is a new competitive SARS-CoV-2 neutralization ELISA test utilizing the same principle as GenScript sVNT (Fig. 1A). The GenScript is a qualitative assay, whereas the Dynamiker assay can be used for either qualitative or quantitative detection of anti-SARS-CoV-2 nAbs^20^. For qualitative result interpretation, percent inhibition was calculated using the same formula as GenScript, where inhibition ≥ 30% was considered positive and < 30% was negative. For quantitative assessment, standards were used to plot a standard curve by logistic regression, and the concentration of nAbs in international units per ml (IU/ml) was calculated using the generated formula. According to the manufacturer's instructions: a concentration of ≥ 20 IU/ml was considered positive, and a concentration < 20 IU/ml was considered negative^20^.

### SARS-CoV-2 NTAb assay

The third assay is an automated competitive binding immuno-enzymatic assay (anti-SARS-CoV-2 NTAb assay) developed by Mindray (catalog No. SARS-CoV-2 Neutralizing Antibody 121) for quantitative detection of nAbs against SARS-CoV-2 RBD. In this assay, anti-SARS-CoV-2 nAbs in the sample compete with ACE2-ALP conjugate for RBD-binding sites (Fig. 1B). The resulting chemiluminescent reaction is measured as relative light units (RLUs) by a photomultiplier built into the system, and the level of nAbs is determined via a calibration curve with a cutoff index of ≥ 10–1000 IU/ml.

#### Preparation of RBD-coated magnetic beads

The carboxylic acid magnetic beads of JSR (CAT: MagnosphereTM MS160/Carboxyl) and homemade RBD (His Tag, expressed by human 293 cells) were used in this study. First, magnetic beads were activated in EDC (1-(3-Dimethylaminopropyl)-3-ethylcarbodliimide hydrochloride) buffer on a roller for 30 min and then collected by the magnetic separation. Secondly, the beads were resuspended in 50 mM MES (2-(N-Morpholino) ethanesulfonic acid hydrate) buffer with RBD and then incubated on a roller for 2 h at room temperature. Lastly, the beads were collected, washed, and resuspended in 50 mM Tris (2-Amino-2-hydroxymethyl-1,3-propanediol) buffer with 0.05% Tween-20 and 0.5% bovine serum albumin (BSA), and stored at 2–8 °C.

#### The preparation of ACE2-alkaline phosphatase (ALP) conjugation

The alkaline phosphatase (ALP) of BBI (CAT: 03535452) and homemade ACE2 (Fc-tag, expressed by human 293 cells) were used for conjugating. ALP and ACE2 were dialyzed in 0.05 M MES, pH 4.5, mixed at a molar ratio of 2 ALP to 1 ACE2, and then EDC and sulfo-NHS (Thermo Fisher) were added to obtain a concentration of 2 mM EDC and 5 mM sulfo-NHS, reacted for 2 h at room temperature. The conjugate was blocked by glycine and purified by gel filtration, stored at 2–8 °C.

#### Assay principle

In the first step, sample (10 µL), sample treatment solution, RBD-coated magnetic beads, and ACE2-ALP conjugate are added into a reaction cuvette. The SARS-CoV-2 NTAbs in the sample competes with the ACE2-ALP conjugate for RBD-binding sites. After binding, the RBD-magnetic beads bound to the NTAbs are magnetically captured, whereas unbound substances are removed by washing. In the second step, the substrate solution is added to the reaction cuvette and catalyzed by ACE2-ALP conjugate in the immunocomplex retained on the magnetic beads. The resulting chemiluminescent reaction is measured as relative light units (RLUs) by a photomultiplier built into the system. The NTAb concentration can be determined via a calibration curve established on an encoded Master Calibration Curve.

#### Calibration curve

This NTAb assay uses a three-point calibration made by SARS-CoV-2 antibodies, which exhibited neutralizing activity by pVNT. The RLUs generated by samples are read by the matching analyzer and transferred to concentration values through the master calibration curve defined by the three-level calibrators. The results are calibrated to the internal standard and shown in the unit of Arbitrary Units per ml (AU/ml), which was converted to international unit per ml (IU/ml).

### Pseudovirus neutralization test (pVNT)

In pVNT, pseudovirus expressing SARS-CoV-2 S protein competes with anti-SARS-CoV-2 nAbs, which block its binding to human ACE2 receptors on the host cell surface (Fig. 1C). Pseudovirus expressing SARS-CoV-2 S protein of SARS-CoV-2 wild-type was prepared using human embryonic kidney (HEK293T) cells (ATCC, USA) infected with vesicular stomatitis virus (VSV) ΔG-luc seed virus as previously described^21,22^. Cells were maintained in Dulbecco's modified Eagle medium (DMEM) supplemented with 10% fetal bovine serum (FBS), 2 mM glutamine, and 1× penicillin/streptomycin in a 37 °C incubator with 5% CO_2_. Briefly, the day before transfection, cells were plated into 10 cm culture plates at a density achieving ~ 80% confluency. On the next day, calcium phosphate transfection reagents (Promega, USA) were used to co-transfect the cells with three plasmids (packaging plasmid pCMVΔR8.2, transducing plasmid pHR' CMV-Luc and CMV/R-SARS-CoV-2 S plasmid) and then incubated overnight. On the next day, the old culture medium was replaced with a fresh medium, and after 48 h, the supernatant was collected, filtered, aliquoted, and frozen at − 80 °C. For the pseudovirus titration assay, HEK293T cells were used, as described by Wang et al.^22^; these cells were kindly provided by the Viral Pathogenesis Laboratory, Vaccine Research Center, National Institute of Health (NIH). Briefly, HEK293T cells were first plated at 1 × 10^4^ cells per well in a 96-well white/black isoplate (PerkinElmer, MA) and cultured overnight. The following day, the culture medium was removed, and two-fold serial dilutions of the pseudovirus were added to the cells and incubated for 2 h. Then, 100 μl of fresh medium was added, and after 72 h, cells were lysed using 1× lysis buffer, and 50 μl of luciferase substrate ((Bio-Glo™ Luciferase Assay System, Promega, USA) was added to each well. Luciferase activity was measured using a luminescence plate reader (Infinite pro200, Tecan).

Following pseudovirus titration, the neutralization assay was carried out as described elsewhere and shown in Fig. 4A^22^. For pVNT, two-fold serially diluted sera samples were mixed and pre-incubated with 1 × 10^6^ RLU of SARS-CoV-2 spike for 30 min at room temperature. Then, the mixture was added to ACE2-transfected HEK293T cells in duplicates and incubated for 2 h, followed by adding 100 μl of fresh DMEM. After 48 h, cells were lysed, and 50 μl of luciferase substrate Promega, USA) was added to each well. Luciferase activity was measured using a luminescence plate reader (Tecan, Switzerland), and percent inhibition (%) was calculated for each sample.

### Statistical analysis

Data were analyzed using GraphPad Prism 9.2.0. (San Diego, CA, USA). Results were plotted as median values with the interquartile range. One-way ANOVA tests were performed to compare the groups, and P-values ≤ 0.05 were considered statistically significant. Correlation and linear regression analysis between log-transformed assay readings were performed the Pearson correlation coefficient was calculated with a 95% confidence interval (95% CI). Correlation interpretation was as follows: a coefficient of 0–0.39 indicates a weak correlation, 0.40–0.59 indicates a moderate correlation, 0.6–0.79 indicates a strong correlation, and 0.8–1 indicates a very strong correlation. In all graphs, significance was *p ≤ 0.05, **p ≤ 0.01, ***p ≤ 0.001, or ****p ≤ 0.0001. Receiving operating characteristic (ROC) curve analysis and Youden index were used to assess the assay thresholds (cutoff) by identifying optimized ones. A nonparametric ROC analysis was performed for each automated immunoassay to estimate the area under the curve (AUC). Statistically, the bigger the AUC, the more accurate a tool is in terms of diagnostic performance. The relation between AUC and diagnostic accuracy applies as follows: an AUC of < 0.5 suggests no discrimination (ability to diagnose patients with and without the disease or condition based on the test), 0.5–0.6 indicates poor discrimination, 0.6–0.7 indicates sufficient discrimination, 0.7–0.8 is considered good, 0.8–0.9 is excellent, and > 0.9 is outstanding^23,24^. The cutoff values for optimal sensitivity and specificity were determined by calculating Youden's index J using the formula: J = sensitivity + specificity − 1. This index is typically used as a summary measure of the ROC curve to help determine the optimal thresholds for each assay and compare it with other tests^25^.

## Results

### Performance assessment of sVNT assays in compassion to PCR

**Figure 2:**
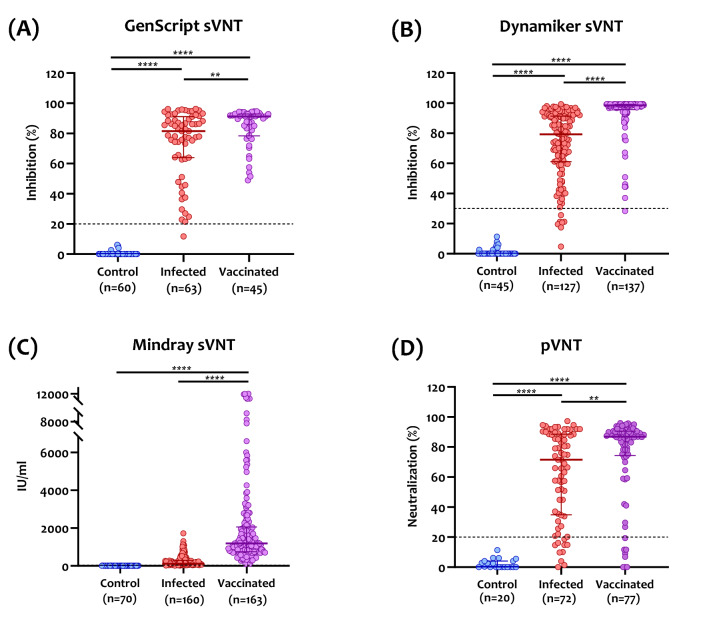
Dot plot of nAb results obtained from (**A**) GenScript SARS-CoV-2 surrogate virus neutralization test (sVNT), (**B**) Dynamiker sVNT, (**C**) Mindray NTAb sVNT, and (**D**) pseudovirus neutralization test (pVNT), using samples collected from healthy controls, SARS-CoV-2 infected patients, and COVID-19 vaccinated individuals. Individual points obtained, median values, and interquartile ranges (IQR) are shown. The dotted lines represent the cutoff at 30% inhibition for GenScript's and Dynamiker's sVNT, 33.1 IU/ml for Mindray's sVNT, and 20% neutralization for pVNT. P values were calculated using one-way analysis of variance (ANOVA) test. *p < 0.05, ***p < 0.001, **** *p* < 0.0001.

The performance assessment and quantitative results for each sVNT assay are shown in Fig. 2. All three assays demonstrated a clear separation between confirmed-positive and confirmed-negative samples, resulting in 100% specificity. GenScript sVNT showed the highest sensitivity (100%) in samples collected from vaccinated individuals and the lowest sensitivity in samples collected from infected patients (96.2%) with a median percent inhibition of 91.2% (interquartile range (IQR): 78.4–92.9%) and 81.6% (IQR: 63.9–91.1%), respectively (Fig. 2A). Both Dynamiker and Mindray sVNTs showed comparable sensitivities, around 97% in infected and vaccinated groups. Median percent inhibition for Dynamiker sVNT of infected patients was lower compared to those of vaccinated individuals, 79.3% (IQR: 61.1–91.3%) and 98.6% (IQR: 95.7–99.2%), respectively (Fig. 2B). Similarly, Mindray sVNT showed median nAb levels of 261.8 IU/ml (IQR: 163–574.6 IU/ml) in infected patients and 335.0 IU/ml (IQR: 169.1–733.7) in vaccinated individuals (Fig. 2C). As for the pVNT, median neutralization was higher in vaccinated individuals [86.7% (IQR: 70.7–90.6)] than infected patients [71.5% (IQR: 34.8–88.5)].

### Correlation with anti-SARS-CoV-2 binding antibodies (bAbs)

**Figure 3:**
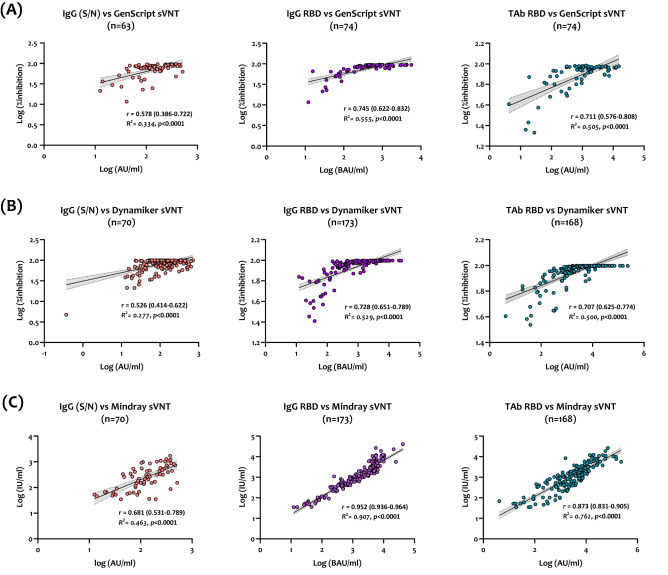
Correlation and linear regression analysis between each sVNT and serological assays detecting anti-SARS-CoV-2 binding antibodies, including anti-S and N IgG antibodies (orange), anti-RBD IgG (violet), and anti-RBD total antibodies (turquoise) for samples collected from previously infected and vaccinated individuals. (**A**) GenScrip sVNT, (**B**) Dynamiker sVNT, (**C**) Mindray NTAb sVNT. Presented data show the log-transformed values of quantitative results obtained by each assay.

Correlation and linear regression analysis between sVNT and serological assays detecting bAbs against SARS-CoV-2 were performed. As shown in Fig. 3A–C, nAbs detected by all sVNT assays demonstrated moderate to strong correlation with bAbs targeting SARS-CoV-2 S and N proteins in sera collected from infected and vaccinated groups (*r* ranging between 0.526 to 0.681 for Dynamiker and Mindray, respectively). Further, all assays strongly correlated with IgG antibodies targeting SARS-CoV-2 RBD; correlation coefficients ranged between 0.728 to 0.952 for Dynamiker and Mindray, respectively. Total antibodies targeting the RBD also denoted strong to excellent correlations with the three sVNTs (*r* ranging between 0.707 to 0.873 for Dynamiker and Mindray, respectively).

### Correlation with pVNT

**Figure 4:**
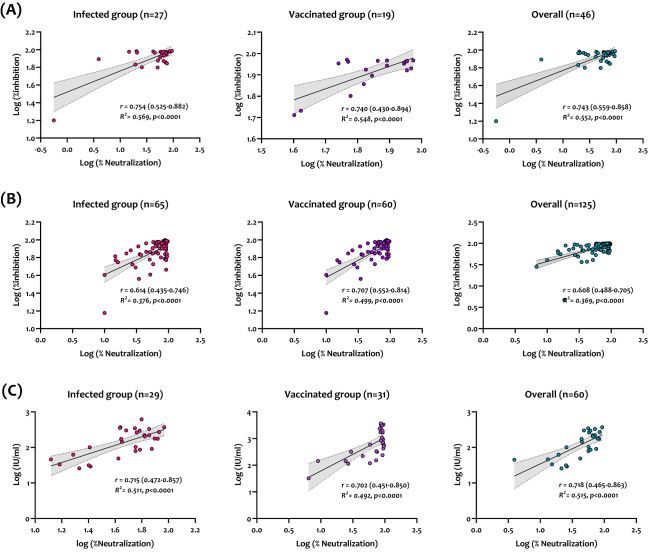
Correlation and linear regression analysis between pVNT and GenScript sVNT (**A**), Dynamiker sVNT (**B**), and Mindray NTAb sVNT (**C**) using samples collected from SARS-CoV-2 infected patients and vaccinated individuals. Correlation and linear regression analyses were performed in GraphPad Prism using Pearson's correlation coefficients (r) and R^2^. Statistical significance was calculated using the two-tailed test. Presented data are the log-transformed values of neutralization (%) by pVNT and nAb results obtained by each sVNT. The dashed lines indicate the 95% confidence intervals of the linear regression plots.

Correlation and linear regression analysis between the three sVNTs and pVNT were performed. As shown in Fig. 4, GenScript demonstrated the strongest correlation with pVNT in the infected group (*r* = 0.754, R^2^ = 0.569), vaccinated group (*r* = 0.740, R^2^ = 0.548), and both groups (*r* = 0.743, R^2^ = 0.552). Also, Mindray and Dynamiker sVNTs showed strong overall correlations with the pVNT in both groups; however, Mindray showed slightly better than Dynamiker (*r* = 0.718, R^2^ = 0.515 vs. *r* = 0.608, R^2^ = 0.369) as shown in Fig. 4B,C.

### Receiving operating characteristic (ROC) curve analysis

**Figure 5:**
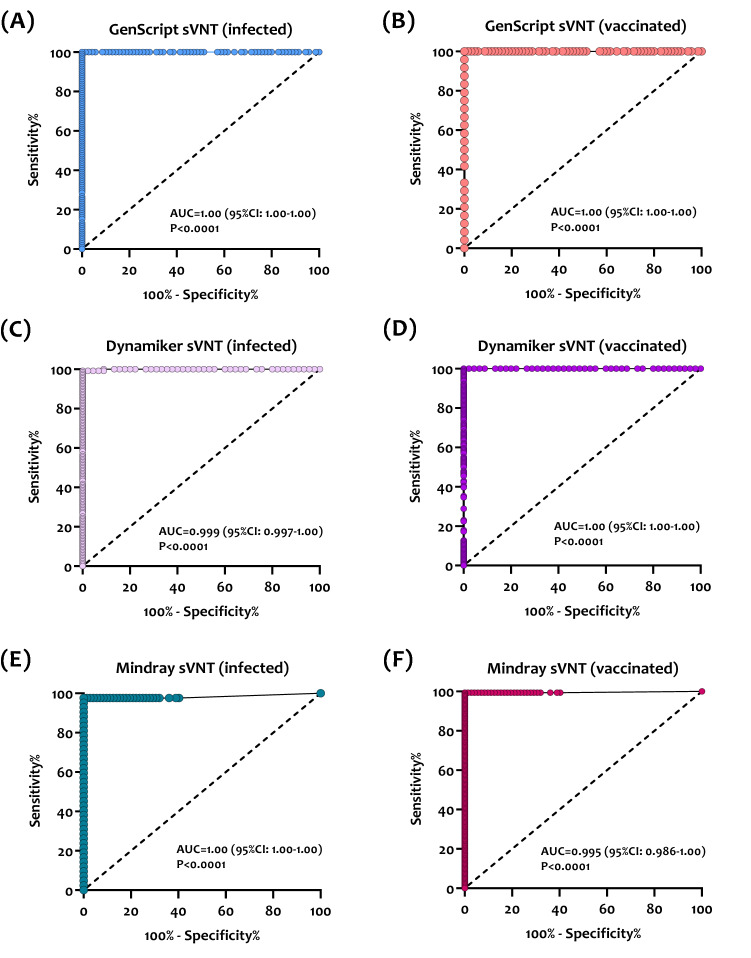
Empirical receiving operating characteristic (ROC) curve analysis for estimating optimal thresholds for sVNT assays targeting nAbs against SARS-CoV-2. (**A**) GenScript sVNT ROC curve in SARS-CoV-2 infected patients (n = 105). (**B**) GenScript sVNT ROC curve in COVID-19 vaccinated individuals (n = 24). (**C**) Dynamiker sVNT ROC curve in SARS-CoV-2 infected patients (n = 127). (**D**) Dynamiker sVNT ROC curve in COVID-19 vaccinated individuals (n = 156). (**E**) Mindray sVNT ROC curve in SARS-CoV-2 infected patients (n = 52). (**F**) Mindray sVNT ROC curve in COVID-19 vaccinated individuals (n = 155). The sensitivity and specificity values correspond to the plotted points which were used to estimate the area under the curve (AUC) and *p*-value for each curve.

The ROC curve analyses were done for the prediction of optimal cutoff values for detecting nAbs in infected patients and vaccinated individuals. The ROC AUCs for the three sVNT assays ranged between 0.99 and 1.0 (*p* < 0.0001), denoting excellent performance for detecting nAbs in both groups (Fig. 5). Using the ROC curves and calculated Youden's index, optimized cutoff values for detecting nAbs by each assay were obtained. As shown in Table 2, the new cutoff indices for detecting nAbs in infected patients were lower than the pre-defined cutoffs provided by the manufacturer of GenScript sVNT (17.1% vs. 30.0%), and Dynamiker sVNT (14.4% vs. 30.0%) and higher for Mindray NTAb sVNT (51.3 IU/ml vs. of 33.1 IU/ml). As for the vaccinated group, new cutoff indices were lower than pre-defined cutoff indices for Dynamiker sVNT (51.3 IU/ml vs. 33.1 IU/ml) and Mindray NTAb sVNT (24.2 IU/ml vs. 33.1 IU/ml) and slightly higher for GenScript sVNT (31.4% vs. 30.0%). Applying these new cutoff values increased the sensitivity of all three assays while maintaining the specificity at 100%, indicating that the cutoff can be adjusted based on the tested cohort without significantly affecting the assay's performance.

**Table 2:** Analytical performance of the evaluated sVNT assays using the pre-defined cutoff values and the newly calculated cutoff values determined using Youden's index.

Assay	GenScript sVNT (cPass)	Dynamiker sVNT	Mindray NTAb sVNT
Pre-defined cutoff	30%	30%	33.1 IU/ml
Sensitivity in the infected group (95% CI)	96.2% (90.6–98.5)	97.0% (94.1–98.5)	97.1% (94.4–98.5)
Sensitivity in the vaccinated group (95% CI)	100% (86.2–100%)	97.0% (94.1–98.5)	97.1% (94.4–98.5)
Specificity (95% CI)	100% (94.8–100)	100% (92.1–100)	100% (94.9–100)
New cutoff for infected group	17.1%	14.4%	51.3 IU/ml
Sensitivity using the new cutoff (95% CI)	100% (96.5–100%)	99.2% (95.7–100)	97.6% (87.7–99.9)
New cutoff for the vaccinated group	31.4%	19.9%	21.4 IU/ml
Sensitivity using the new cutoff (95% CI)	100% (86.2–100%)	100% (97.6–100)	99.4% (96.4–100)
Specificity using the new cutoff (95% CI)	100% (94.8–100)	100% (92.1–100)	100% (95.0–100)

## Discussion

Since the COVID-19 pandemic, a consensus has emerged that serological assays provide an essential tool in the pandemic response. However, inadequate performance data in some early surveys and gaps in immunological knowledge hindered the agreement on selecting reliable immunoassays^26^. Also, there is a lack of information about the functional activity of SARS-CoV-2 antibodies that may be associated with protective responses. For these reasons, the MNA and pVNT are still considered the gold standard to counteract viral infection. To bypass the intense work of and time-consuming cell-culture-based neutralization assays, sVNT has been developed to provide a rapid, accurate, and large-scale alternative^27^. Here, we assessed the performance of three different sVNTs using the same concept: nAbs in a particular sera sample would disrupt the binding between viral RBD and its ACE2 receptor in either a solid or liquid phase platform. In addition to evaluating the GenScript assay, which has been characterized by several studies^3,7,27–30^, we assessed two new surrogate assays: Dynamiker ELISA-based assay^20^ and an automated liquid phase assay Mindray NTAb^31^.

Unlike previous studies assessing sVNT assays using only infected patients' sera, we characterized the three assays using sera collected from infected and double vaccinated (BNT162b2 or mRNA-1273) individuals. The performance of the assays was correlated with the gold-standard pVNT and serological assays measuring anti-SARS-CoV-2 bAbs. Overall, all three sVNTs demonstrated robust performance in terms of sensitivity and specificity (Fig. 2 and Table 2). Also, all sVNT assays, especially Mindray NTAb, showed the best correlation with IgG antibodies targeting the RBD. This is most likely because IgG antibodies comprise most of the antibody isotypes that target the RBD^32^. Further, GenScript cPass sVNT exhibited the best linear correlation with pVNT in samples collected from both infected and vaccinated individuals. These results are consistent with previous studies assessing the performance of GenScript sVNT compared to MNA and pVNT, demonstrating high specificity and even more sensitivity^7,30,33,34^. The two other assays, Dynamiker and Mindray NTAb, also exhibited a significant linear correlation with pVNT.

Notably, all assays showed higher median neutralization in the vaccinated group than in the infected group (Fig. 2). These findings align with previous studies reporting higher nAb titers induced by mRNA COVID-19 vaccines compared to those induced by natural infection^35,36^. This could be attributed to the high doses used for mRNA vaccines: 100-μg dose mRNA-1273 and 30-μg dose for BNT162b2 vaccine^37^. In addition, a study reported that mRNA-1273 elicits an RBD-focused nAb response that targets a broader range of epitopes than those produced by natural infection, suggesting another possible reason for this difference^38^.

Previous studies reported that nAbs could target non-RBD domains on the S protein, including the N-terminal domain (NTD) and S2 subunit^39^. Therefore, it is presumed that specific immunoassays directed only against the RBD might underestimate protective SARS-CoV-2 neutralization titers, especially in cross-reactive samples. However, studies have shown that anti-RBD antibodies comprise around 90% of total neutralizing activity^40–42^. This is supported by our finding, where we have demonstrated a strong correlation between the assessed sVNTs and neutralizing titers targeting the S protein by pVNT.

Another key advantage offered by these sVNTs over most ELISA or point-of-care serological tests is their ability to detect total nAbs in an isotype-independent manner, increasing test sensitivity. A study assessing SARS-CoV-2 antibody responses against the RBD showed that robust neutralization was associated with high-titer, multi-isotype antibody responses, with IgG showing the strongest correlation, followed by IgA and IgM^43^. This suggests that RBD-based sVNT assays may benefit from detecting additional isotypes, rendering them better suited as predictors for neutralizing activity.

Although the GenScript assay is not considered suitable for quantitative analysis of nAbs, the strong correlations observed between the assay with pVNT and quantitative assays measuring bAbs suggest that it could be utilized for this purpose if used with appropriate standards. The Dynamiker sVNT, however, could be used for both qualitative and quantitative assessment of nAbs along with Mindray NTAb, which provides a quantitative measurement for nAbs. Hence, quantitatively employing these assays is likely to be more informative, particularly if sequential samples from individuals are collected to monitor the decline in nAb responses following infection and/or vaccination. In addition, ROC curve analysis showed that cutoff values for nAbs capable of conferring immunity following SARS-CoV-2 infection and vaccination could be adjusted depending on the tested cohort without affecting the performance or accuracy of the assays. Thus, applying these new cutoffs could help assess the immunization status and identify subjects at risk of infection or reinfection. Also, it could be incorporated into future assay standardization and quality control.

One major concern remains, how would the new variants of concern (VOCs) affect the performance of these assays, given that most mutations found in these variants are in the S protein, specifically the RBD. Based on the data obtained from previous studies, significant reductions in neutralization against the VOCs compared to the ancestral Wuhan-Hu-1 strain were reported in sera from fully vaccinated individuals and recovered COVID-19 patients^44^. Moreover, limited variant-specific cross-neutralizing immunity was observed after either Delta or Omicron breakthrough infections, suggesting that these variants are less immunogenic than previous VOCs^45^. Accordingly, neutralization assays utilizing the ancestral strain antigens are expected to underestimate the neutralizing immunity in vaccine breakthrough infections.

In conclusion, solid-phase and liquid-phase sVNTs targeting anti-SARS-CoV-2 RBD nAbs could be considered valuable, robust, and high-throughput alternatives for cell-based neutralization assays. This will enable the characterization of immune response and durability in infected and vaccinated individuals. Also, the ability of these assays to be automated and performed rapidly renders them highly potent diagnostic tools for the ongoing monitoring of functional immune responses at both the individual and population levels.

## Data Availability

All relevant data are available within the manuscript.
